# Disparities in Paediatric Injury Mortality between Aboriginal and Non-Aboriginal Populations in British Columbia, 2001–2009

**DOI:** 10.3390/ijerph13070651

**Published:** 2016-07-07

**Authors:** Ofer Amram, Blake Byron Walker, Nadine Schuurman, Ian Pike, Natalie Yanchar

**Affiliations:** 1 Department of Geography, Simon Fraser University, 8888 University Drive, Burnaby, BC V5A 1S6, Canada; oamram@sfu.ca (O.A.); nadine@sfu.ca (N.S.); 2 Department of Pediatrics, Faculty of Medicine, University of British Columbia, Vancouver, BC V6H 3V4, Canada; ipike@cw.bc.ca; 3 BC Injury Research and Prevention Unit, Child and Family Research Institute, BC Children’s Hospital, Vancouver, BC V5Z 4H4, Canada; 4 Department of Surgery, Dalhousie University, Halifax, NS B3H 2Y9, Canada; natalka1963@gmail.com

**Keywords:** paediatric injury, injury mortality, Aboriginal population

## Abstract

Injury is the leading cause of death among children and youth in Canada. Significant disparities in injury mortality rates have been observed between Aboriginal and non-Aboriginal populations, but little is known about the age-, sex-, and mechanism-specific patterns of injury causing death. This study examines paediatric mortality in British Columbia from 2001 to 2009 using comprehensive vital statistics registry data. We highlight important disparities in Aboriginal and non-Aboriginal mortality rates, and use the Preventable Years of Life Lost (PrYLL) metric to identify differences between age groups and the mechanisms of injury causing death. A significantly greater age-adjusted mortality rate was observed among Aboriginal children (OR = 2.08, 95% CI: 1.41, 3.06), and significantly higher rates of death due to assault, suffocation, and fire were detected for specific age groups. Mapped results highlight regional disparities in PrYLL across the province, which may reflect higher Aboriginal populations in rural and remote areas. Crucially, these disparities underscore the need for community-specific injury prevention policies, particularly in regions with high PrYLL.

## 1. Introduction

Injury is the leading cause of death for children above the age of five worldwide, and above the age of one in Canada [1,2,3]. Over the last five decades, there has been a 50% decline in childhood injuries within Canada. However, while the absolute number of injuries has declined, several studies have demonstrated that the prevalence of injury remains substantially higher among socioeconomically deprived populations, and that this disparity is increasing [3,4,5,6,7,8].

Most evident in the Canadian context is the socioeconomic gradient of injury mortality and morbidity rates between Aboriginal (those who self-identify as Inuit, Metis, or First Nations) and non-Aboriginal populations. Canadian Census data estimate that 4.3% of the population is Aboriginal (2011 data) [9]. However, injuries and other external causes of death account for over half of all Potential Years of Life Lost (PYLL) among Aboriginal populations in Canada [10]. Lower life expectancies have resulted in a younger population distribution, with 28% of the Aboriginal population aged 14 or younger, compared to 16.5% of the non-Aboriginal population [10]. This uneven population distribution, in conjunction with a greater burden of injury among Aboriginal children and youth, accounts for the observed disparities in PYLL [4,11,12,13,14,15]. There is a strong body of evidence that Aboriginal populations experience greater rates of violent injury and suicide than non-Aboriginal groups. For example, a 2010 report of First Nations populations in Ontario found an elevated rate of intentional (2.7 times greater) and self-inflicted (4.6 times greater) injury, compared to the non-Aboriginal population [16]. However, in contrast to the socioeconomic trends in injury, the gap between Aboriginal and non-Aboriginal injury rates is reportedly closing, with a greater decrease in injury among Aboriginal children from 1986 to 2010 [13]. While the trends in Aboriginal and non-Aboriginal injury risk have been thoroughly reported in previous studies, less is known about the mechanisms of injury. A report published a decade earlier by Health Canada on the mechanisms of injury-related death found higher mortality rates among Aboriginal populations due to suicide, fire, drowning, and motor vehicle collisions [10]. However, age-specific differences in the mechanism of injury between Aboriginal and non-Aboriginal populations are yet to be reported, and may provide useful information for community-targeted injury prevention policy and practice.

This study therefore seeks to identify more nuanced patterns of child and youth injury death by examining individual patient-level data by age, sex, Aboriginal status, and mechanism of injury for the study period from 1 April 2001 to 31 March 2010 (corresponding with the BC Vital Statistics calendar year). While the few previous studies that do exist provide relatively consistent mortality and morbidity estimates, this study is unique in that it uses a Preventable Years of Life Lost (PrYLL) metric for quantifying and comparing the impact of injury on Aboriginal and non-Aboriginal populations. PrYLL is the number of life-years lost due to preventable causes, as opposed to all-cause mortality and has been used in several previous injury studies [17,18]. This metric is particularly useful for quantifying the societal burden of child and youth injury and highlighting opportunities for prevention policy and practice. In order to identify regions where targeted injury prevention policy and practice may drive the greatest overall reductions in child and youth mortality, we use geographical information systems to map PrYLL across the study area; to our knowledge, this constitutes an innovative and novel approach. Mapping technologies have been previously used in health research to identify and highlight areas where health disparities occur [19,20]. They also have been proven to be a powerful tool for the analysis and dissemination of such information [21].

## 2. Data and Methods

The study protocols received approval from the Research Ethics Board at Simon Fraser University and Population Health Data British Columbia (BC) prior to analysis.

Patient mortality data were acquired through Population Health Data BC, comprising vital statistics data collected by the Vital Statistics Agency of BC. Included were all cases in the BC where a patient aged 0 to 15 years expired as a direct result of injury from 1 April 2001 to 31 March 2010, inclusive. In addition, the postal code location at which the injury occurred was included, enabling the mapping of study results. Canadian postal codes are classified as urban or rural; we used this classification to assist in the description of mapped results and refer to remote regions as those that do not contain a town or city.

Demographic variables included in this analysis were: patient age groupings 0–4, 5–9, and 10–15 years, sex, and Aboriginal status. Age groups were selected for consistency with the relevant governmental reporting and the epidemiological literature. The mechanism of injury causing death was coded at the time of patient intake according to the International Classification of Diseases version 10 codes (ICD-10) [22]. Mortality cases were tabulated and summarised according to the variables described above. Selected variables were cross-tabulated and categorical differences were examined using Pearson’s chi-square and Cochran-Armitage chi-square where appropriate.

Risk population denominators for age-specific and age-adjusted mortality rate calculations were acquired from the Statistics Canada 2006 Census of Population and are publicly available online [23]. The census year 2006 was selected as it was the approximate midpoint of the study period. Population counts for Aboriginal and non-Aboriginal populations were tabulated by age groupings 0–4, 5–9, and 10–14 years. The population age 15 is available for the general population, but not specifically for the Aboriginal population. An estimate of the Aboriginal population age 15 was therefore estimated to be equal to the 15 year-old proportion of the non-Aboriginal population ages 15–19.

Age-specific mortality rates were calculated for the three patient age groupings (0–4, 5–9, 10–15) by sex and Aboriginal status, using the population denominators described above. Age-specific mortality rates by age group and Aboriginal status were also calculated for each mechanism of injury, and odds ratios with 95% CI were estimated using Fisher’s exact confidence intervals. The 2006 BC standard population for each age group was used to age-standardise mortality rates for Aboriginal and non-Aboriginal populations. Significance of differences with 95% CI between Aboriginal and non-Aboriginal rates were calculated. All statistical calculations were conducted using WinPepi version 11.42 [24].

Preventable Years of Life Lost (PrYLL) was calculated for each patient based on a reference life expectancy of 80 years for all populations and tabulated for Aboriginal and non-Aboriginal patients across the nine-year study period [17]. PrYLL per capita were then calculated for each 2006 census dissemination area and mapped using geographical information systems. The Local Getis-Ord Gi algorithm was used to compute, map, and detect statistically significant (*p* < 0.05) spatial clusters of PrYLL per capita across the province [25]. Getis G is a function that is commonly used to measure and examine the relationship of spatially distributed variables and it also provides a way to map and visualize the clusters over space [25].

## 3. Results

A total of 334 deaths occurred during the study period. A greater number of paediatric injury deaths occurred among males in all three age groups (Table 1), and while the difference was larger among older age groups, this was not a statistically significant difference (Cochran-Armitage χ^2^ = 2.611, *df* = 1, *p* = 0.106). Similarly, a greater number of male deaths were observed for both Aboriginal and non-Aboriginal mortality, though the male-female difference was greater for the non-Aboriginal group. However, this association was not statistically significant (Pearson’s χ^2^ = 0.338, *df* = 1, *p* = 0.561).

Age-specific mortality rates were observed to be consistently higher among Aboriginal populations for all three age groups, though the odds difference decreased among the oldest children, as shown in Table 2. Odds ratios for Aboriginal versus non-Aboriginal injury mortality were statistically significant for all age groups. When age-adjusted to the 2006 BC standard population, Aboriginal mortality odds were approximately twice those of non-Aboriginal children, a statistically significant difference.

**Figure 1:**
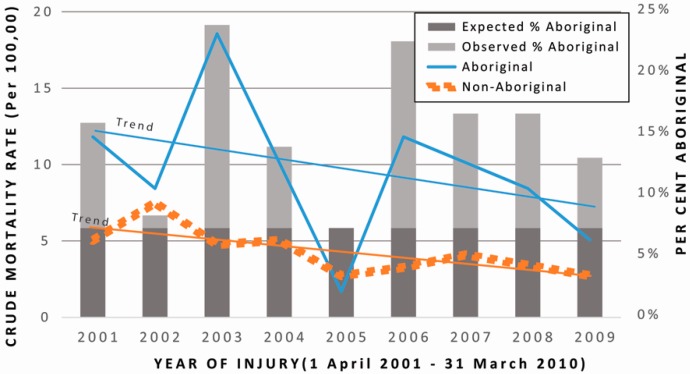
Temporal trends in the crude mortality rates for Aboriginal (**Blue** line) and non-Aboriginal (**Orange** line) children and youth ages 0–15, with respective trendlines, and proportion of total deaths among Aboriginal children, from 2001 to 2009.

Over the study period from 1 April 2001 to 31 March 2010, declines in paediatric mortality were observed for both Aboriginal and non-Aboriginal populations, as shown in Figure 1. Results of the Cochran-Armitage χ-square test (χ^2^ = 0.149, *df* = 1, *p* = 0.699) indicate that the trend in mortality rates did not significantly differ between Aboriginal and non-Aboriginal children. However, the proportion of Aboriginal children among decedents was higher than expected throughout the time period, except in the years 2002 and 2005 (represented by bars in Figure 1). The lack of cases in 2005 is most likely the result of random variation.

Differences in mortality odds between Aboriginal and non-Aboriginal populations were found to vary by mechanism of injury causing death, as shown in Table 3. Significantly greater mortality risk among Aboriginal children was identified for assault and suffocation among children ages 0–4, and fire among children ages 5–9. The greatest risk difference was observed for children ages 0–4 for suffocation, with over 8 times the risk of death among Aboriginal children. Notably, an elevated (but not statistically significant at the 95% confidence level) risk was observed for vehicular cause of death among those ages 5–9 and 10–15, and assault among children ages 10–15. A low number of cases limited inferential power, although large odds ratios were observed. All-cause mortality odds were significantly higher among Aboriginal children for all three age groups.

A total of 23,468 preventable years of life were lost due to paediatric trauma during the study period, as shown in Table 4 Vehicular death, assault, pedestrian trauma, and other causes were the greatest contributors to total life years lost. While absolute numbers of preventable years of life lost (PrYLL) are lower among Aboriginal populations, the nine-year rate is higher for both male and female Aboriginal groups. The highest rates of life years lost were among Aboriginal children, resulting from vehicular and assault injuries.

**Figure 2:**
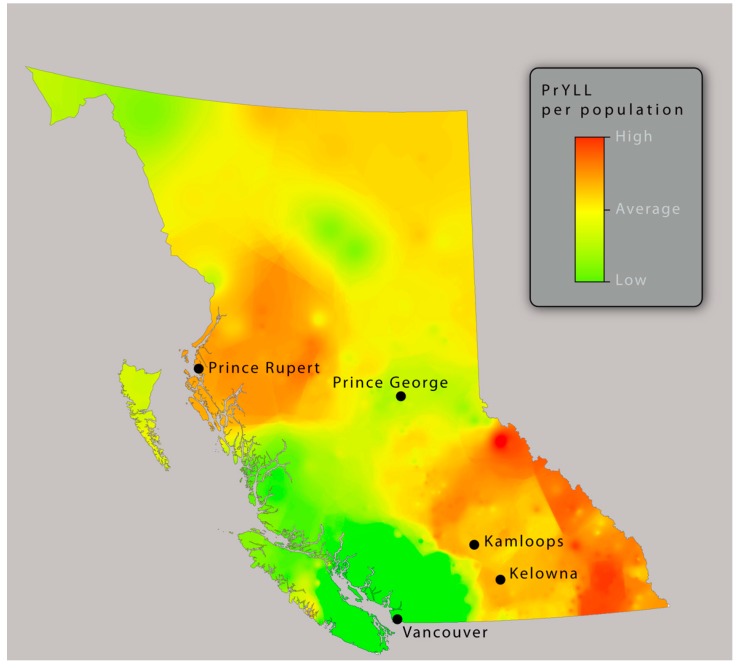
Spatial clustering of PrYLL per capita. Clusters of high PrYLL are shown in red, and tend to occur in rural and remote regions.

When mapped, PrYLL per capita varies geographically across BC, as shown in Figure 2. Clusters are observed in the regions with predominantly rural-classified postal codes, in the mountainous regions east of Kelowna and Kamloops, and in a remote region northwest of Prince George. Relatively low PrYLL occurred in and around the urban regions of Vancouver and the adjacent Vancouver Island. These findings are suggestive of an urban-rural divide in paediatric injury and highlight areas where targeted interventions may have the greatest impact.

## 4. Interpretation

The results of this study are largely consistent with previous reports [10], in that rates of injury-related mortality were consistently higher among Aboriginal populations than non-Aboriginal populations. Within the study area, the map highlighted important regional differences in the burden of injury as measured by PrYLL. Rural and remote regions were observed to have higher rates of PrYLL per capita, suggesting a disproportionately high burden among these populations. Importantly, Aboriginal populations in Canada are greater in rural and remote regions, and this may be reflected in our results [26]. However, we did not publish Aboriginal status-specific maps in order to preserve patient confidentiality. This study highlights a need for geographically-focussed prevention strategies to narrow the paediatric injury mortality gap between Aboriginal and non-Aboriginal children and youth in BC. Of particular concern are the disparities in mortality rates among victims of assault ages 0–4 years. Within this group, Aboriginal children have nearly six-times the mortality rate of their non-Aboriginal peers. The intentional nature of these injuries underscores a need for culturally-specific interventions tailored to the affected communities. Additionally, the odds of suffocation was over 8 times greater for Aboriginal children ages 0–4. These cases are categorised as non-intentional, which may suggest a need for further research on domestic risk factors such as sleeping spaces, toys and small objects, and first aid ability. The observed geographical disparities in PrYLL rates across the province underscore this need and should support regional and local injury prevention programmes. Crucially, research demonstrates that rural and remote populations have poorer access to trauma care than their urban counterparts [27]. This disparity is directly reflected in higher rates of trauma-related mortality among rural and remote populations. That Aboriginal children are more likely than non-Aboriginal children to live in rural and remote areas is almost certainly an underlying driver of the observed disparities in this study.

However, the magnitude of differences observed was not as great as reported in some Canadian studies. For example, Oliver and colleagues observed a rate five times greater among Aboriginal children and youth mortality ages 1 to 19 in Inuit regions of Northern Canada, and another study in Newfoundland and Labrador observed mortality rates up to eight times higher; conversely, our study found rates only 2.08 times higher [12,14]. We hypothesise that this observed difference may be due to geographical differences in exposure to risk factors between Northern and Southern communities, but further research will be required to interrogate this. A comparison of mortality rates between Western Canadian provinces highlighted important geographical disparities, where BC was observed to have the highest rate (185 per 100,000), compared to Saskatchewan (142), Manitoba (117), and Canada-wide (28) [10].

Temporally, our findings are congruent with those of George and colleagues, in that we observed a closing gap between Aboriginal and non-Aboriginal children and youth injury, though our study focused only on mortality and the resulting rates were unstable due to a small Aboriginal denominator population [13]. Despite an overall decrease in mortality rates due to injury, the proportion of Aboriginal cases remained generally consistent across the study period, with some temporal variability observed.

This study found differences in age- and mechanism-specific mortality rates between Aboriginal and non-Aboriginal children and youth, though a low number of overall cases limited the power of the study to demonstrate statistical significance in most categories. A Health Canada report found that crude rates of death due to drowning were found to be one order of magnitude greater among Aboriginal children under the age of five, compared to non-Aboriginal children of the same age. This disparity was lower for children ages 5 to 14, and while Aboriginal female rates of drowning remained similar to non-Aboriginal rates for ages 15 and older, the Aboriginal male rate increased to nearly 8 times the non-Aboriginal rate ages 15 and older. Conversely, our study found higher rates among Aboriginal youth ages 5–15, but not for children under age 4; none of these odds ratios were statistically significant, it was likely due to small numbers. Previous studies have suggested higher odds of Aboriginal death by drowning due to an increased exposure to water, particularly on Canada’s coasts and on surface ice in rural and remote regions [10].

A study conducted in 2010 found significantly greater mortality rates among Aboriginal children due to burns [12]. Similarly, we observed a significantly greater rate of fire-related death among Aboriginal children ages 5–9, but were unable to detect significant differences among other age groups due to a small number of cases in these categories. However, a 2012 study suggests that this observed disparity among Aboriginal populations may be due to housing structures more vulnerable to fire, compounded by higher smoking rates and an underuse of smoke detectors in the home [28]. Accordingly, injury prevention strategies should emphasize fire risk in the home, the links between smoking and fire risk, and how to minimize fire risk. In addition, a policy intervention at a local level to increase smoke detector use in the home may reduce fire-related child mortality.

Deaths due to suffocation were observed to be highest among Aboriginal children ages 0–4. Suffocation deaths are known to be preventable, and are often caused by unsafe sleeping and playground environments [29]. Safety programmes and parent education are an effective means of reducing suffocation-related death, and provide important opportunities for reducing infant mortality in our study area [30].

Similar to previous studies, we found significantly higher rates of child death 0–4 years due to assault among Aboriginal populations. There is a growing body of research from Canada, Australia, and New Zealand suggesting the need to explore culturally appropriate alternatives to the current justice systems in place [7,8,31,32,33]. However, more important are interventions that target the underlying drivers of violence against children [34,35,36].

## 5. Limitations

The lack of sex-specific age distributions and life expectancies for Aboriginal populations may have induced minor degrees of error in the calculation of ASMR and AAMR. However, we believe the estimation method for Aboriginal persons ages 10–15 to be adequately accurate for this purpose. Importantly, this study was limited in its power to attain statistical significance due to low numbers of paediatric trauma deaths, resulting in an observed instability in the overall decline in mortality rates among Aboriginal children. However, these data are a population and so all the observed disparities represent real differences in mortality and must therefore be considered in their translation for injury prevention policy and practice.

## 6. Conclusions

This study provides an important analysis of paediatric injury mortality in BC and highlights differences between age groups, mechanisms of injury, and Aboriginal/non-Aboriginal status. PrYLL provides a useful metric by which to quantify, compare, and map the impact of injury mortality, and highlight subpopulations and regions for targeted prevention efforts. Crucially, we provide additional and detailed evidence of a persistent racial disparity in child and youth mortality and call for community-specific and geographically-focused injury prevention programmes to narrow this gap and reduce paediatric injury death across all populations.

## Figures and Tables

**Table 1:** Number of injury deaths by age, sex, and Aboriginal status.

	Age	Female	Male	Total
**Age (Years)**	0–4	32	40	72
	5–9	22	43	65
	10–15	65	132	197
**Aboriginal Status**	Aboriginal	20	31	51
	Non-Aboriginal	99	184	283
		119	215	334

**Table 2:** Nine-year age-specific mortality rates (ASMR) and age-adjusted mortality rates (AAMR) per 100,000 population with odds ratios and 95% confidence intervals for Aboriginal and non-Aboriginal populations.

	Age	Aboriginal	Non-Aboriginal	OR (95% CI)
ASMR	0–4	10.03	3.97	2.53 (1.39, 4.61)
	5–9	9.02	2.97	3.04 (1.65, 5.57)
	10–15	12.48	7.50	1.66 (1.09, 2.53)
AAMR		10.77	5.17	2.08 (1.41, 3.06)

**Table 3:** Age- and mechanism-specific mortality rates (per 100,000) with odds ratios and 95% CI for decedent age groups 0–4, 5–9, and 10–15 years. Asterisks indicate statistically significant odds ratios.

Cause of Injury (ICD-10)	ASMR/100,000								
	Age 0–4 Years			Age 5–9 Years			Age 10–15 Years		
	Abor.	Non-Abor.	OR (95% CI)	Abor.	Non-Abor.	OR (95% CI)	Abor.	Non-Abor.	OR (95% CI)
Suffocation (W75-84)	24.70	2.97	8.31 (2.35, 29.45) *	5.55	3.17	1.75 (0.04, 13.63)	3.99	1.92	2.08 (0.05, 17.11)
Assault (X85-Y09)	24.70	4.46	5.54 (1.25, 19.86) *	5.55	3.17	1.75 (0.04, 13.63)	27.95	12.51	2.24 (0.84, 5.06)
Drowning (W65-74)	6.17	6.93	0.89 (0.02, 5.85)	5.55	1.81	3.06 (0.06, 30.96)	3.99	2.57	1.56 (0.04, 11.61)
Fire (X00-09)	6.17	1.49	4.16 (0.08, 51.76)	16.66	1.36	12.25 (1.64, 91.61) *	0.00	2.89	
Other/Unknown (Y34)	6.17	4.46	1.39 (0.03, 10.00)	5.55	2.27	2.45 (0.05, 21.91)	23.96	11.87	2.02 (0.85, 4.78)
Pedestrian (V01-09)	12.35	2.97	4.16 (0.41, 23.24)	5.55	3.62	1.53 (0.03, 11.43)	3.99	5.13	0.78 (0.02, 5.01)
Vehicle (V10-99)	0.00	5.94		27.76	8.16	3.40 (0.99, 9.52)	35.94	18.28	1.97 (0.86, 4.00)
All-Cause	80.27	29.23	2.75 (1.38, 5.07) *	72.18	23.56	3.06 (1.53, 5.71) *	99.84	55.16	1.81 (1.14, 2.77) *

**Table 4:** Total Preventable Years of Life Lost (PrYLL) and the PrYLL per 1000 persons (all ages), based on an 80 year reference life expectancy, by sex, mechanism of injury, and Aboriginal status. Denominator populations are sex- and Aboriginal status-specific.

Cause of Death	Total PrYLL			PrYLL Rate per 1000		
	Abor.	Non-Abor.	Both	Abor.	Non-Abor.	Both
Female	1376	7377	8753	23.23	10.93	11.92
Male	1855	12,855	14,710	31.31	19.04	20.03
Suffocation	456	1386	1842	6.99	2.07	2.47
Assault	843	3789	4632	12.95	5.75	6.33
Drowning	213	1909	2122	3.04	2.86	2.87
Fire	296	1058	1354	4.52	1.60	1.83
Other/Unknown	559	3523	4082	8.08	5.30	1.05
Pedestrian	296	2123	2419	4.52	3.22	3.33
Vehicle	969	6048	7017	14.43	9.17	9.59
	3632	19,836	23,468			
